# A Sweet Paradox: Severe Insulin Resistance and Hyperglycemia in Asymptomatic COVID-19 Infection

**DOI:** 10.7759/cureus.40477

**Published:** 2023-06-15

**Authors:** Bhanvi P Ramchandani, Misbah F Azmath, Snigdha R Bendaram, Faryal S Mirza

**Affiliations:** 1 Internal Medicine, University of Connecticut School of Medicine, Farmington, USA; 2 Endocrinology and Metabolism, University of Connecticut School of Medicine, Farmington, USA

**Keywords:** coronavirus disease 2019, sars-cov-2 infection, intravenous insulin, type 1 diabetes mellitus, insulin resistance, asymptomatic covid-19, hyperglycemia

## Abstract

There is a well-established association between hyperglycemia and severe coronavirus disease 2019 (COVID-19) infection, regardless of the diagnosis of diabetes prior to the infection. However, it is unusual for patients with a mild infection to present with severe hyperglycemia and insulin resistance requiring intravenous insulin therapy. Uncontrolled hyperglycemia is associated with worse outcomes in COVID-19, making it crucial to achieve optimal glycemic control, which occasionally requires IV insulin therapy. We report a patient with type 1 diabetes mellitus (T1DM), on hemodialysis, who presented with diabetic ketoacidosis (DKA) due to non-adherence to insulin. He was found to be incidentally positive for COVID-19 on admission. Although he was asymptomatic and did not require steroids for the treatment of COVID-19, he was noted to have persistent severe hyperglycemia requiring unusually high levels of intravenous insulin. This proposes that even a mild infection with severe acute respiratory syndrome coronavirus 2 (SARS-CoV-2) infection can trigger a systemic response that can lead to downstream manifestations including insulin resistance and severe hyperglycemia. Interestingly, our patient had three admissions within the past six months as well as another admission two weeks after the current presentation with DKA secondary to insulin non-compliance, all of which required IV insulin for <24 hours following which he was transitioned to a basal-bolus insulin regimen with well-controlled glucose levels.

## Introduction

Diabetes is a complex chronic metabolic disease that includes type 1 diabetes mellitus (T1 DM) characterized by autoimmune β-cell destruction, leading to absolute insulin deficiency, and type 2 diabetes mellitus, characterized by β-cell dysfunction in a background of insulin resistance [1]. A novel coronavirus, severe acute respiratory syndrome coronavirus 2 (SARS-CoV-2) causing coronavirus disease 2019 (COVID-19) was first reported in Wuhan, China, in December 2019 and since then has infected millions of individuals globally. 

COVID-19 is known to precipitate new-onset hyperglycemia, cause worsening of pre-existing diabetes, or even lead to hyperglycemic emergencies. Prior reports have correlated the degree of hyperglycemia to the severity of the COVID-19 infection since elevated levels of glucose induce viral replication with resultant cytokine production and T-cell dysfunction.

Most cases with severe insulin resistance have been reported in individuals with severe COVID-19 infection and rarely with asymptomatic COVID-19 infection. Patients with underlying diabetes with COVID-19 infection require close monitoring since even a mild infection can cause severe hyperglycemia necessitating the need for IV insulin therapy.

## Case presentation

A 48-year-old male with a past medical history significant for T1DM, end-stage renal disease (ESRD) on hemodialysis (HD), and a previous cerebrovascular accident was admitted to the hospital after refusing insulin therapy and HD at his skilled nursing facility. He also incidentally tested to be positive for COVID-19 on admission, although he was asymptomatic. 

On initial presentation, he was hypothermic with a temperature of 96.1 F, tachycardic with a heart rate of 102 beats/min, and hypotensive with a blood pressure of 96/74 mmHg. He was saturating well on room air and did not require supplemental oxygen. Laboratory studies were remarkable for an initial blood glucose of 932 mg/dl, arterial pH of 7.2, serum bicarbonate of 11 mmol/l, lactic acid of 2.9 mmol/l, elevated beta-hydroxybutyrate at 5.8 mmol/and an anion gap of 27 mmol/l supporting the diagnosis of diabetic ketoacidosis (DKA). His glycated hemoglobin (HbA1c) was 7.9%.

He was admitted to the intensive care unit and started on IV insulin therapy (IVIT). He was initially dialyzed for two consecutive days after admission and was then switched to an alternate-day dialysis schedule. Metabolic acidosis improved dramatically after dialysis with normalization of the blood sugars. He was transitioned to subcutaneous (SQ) insulin the following morning. However, that evening, his blood glucose started trending up and was as high as 500 mg/dl, which necessitated reinstatement of the IVIT. Hyperglycemia continued to worsen despite IV insulin infusion, which was titrated up to 24 units/hr on day 4 of admission. The results of investigations done on day 5 of admission are given in Table 1.

**Table 1:** Results of investigations done on day 5 of admission LDH: lactate dehydrogenase; ESR: erythrocyte sedimentation rate; CRP: C-reactive protein; IGF-1: insulin-like growth factor 1

	Parameter	Lab Value	Normal Range
Insulin and Glucose Tests	Serum glucose	202 mg/dl	75-200 mg/dl
	Random insulin level	33uIU/ml	
	Insulin level in IV infusion bag	669747 uIU/ml	
	C-peptide	<0.1 ng/ml	0.5-3.3 ng/ml
	Insulin antibody	2.5 U/ml	0-0.4 U/ml
Inflammatory Markers	Ferritin	3764 ng/ml	16-336 ng/ml
	Fibrinogen	411 mg/dl	150-400 mg/dl
	LDH	186 U/L	125-220 U/L
	ESR	71 mm	0-15 mm
	CRP	13.3 mg/l	<8 mg/l
	D-dimer	231 ng/ml	0-243 ng/ml
Counter-regulatory Hormones	Cortisol (7:30 AM)	40.6 ug/dl	7-23 ug/l
	IGF-1	87 ng/ml	69-224 ng/ml
	Growth hormone	8.9 ng/ml	0.05-3 ng/ml

No external source of hyperglycemia was identifiable; the patient was afebrile and with normal white cell count and on a strict calorie-controlled diet. He did not require steroids for the treatment of COVID-19. The nurse confirmed good IV access and the IV insulin infusion fluid from the bag was sent for analysis which later showed that insulin was present in the infusion bag. Inflammatory markers checked at the time he was on high dose IVIT showed high levels of cortisol, ferritin, growth hormone, C-reactive protein, and sedimentation rate suggesting an inflammatory response. Insulin antibodies were also present. D-dimer was normal, and fibrinogen was only mildly elevated.

On day 4, he received almost 500 units of insulin in 24 hours while his daily requirements prior to coming to the hospital was around 30-50 units. There was concern that he may be metabolizing IV insulin at a rapid rate; hence, after six hours of receiving IVIT at 24 U/hour, he was given a relatively higher dose of SQ-insulin with insulin glargine (Lantus®) 20 units and insulin lispro 30 units on day 5, in addition to continued IVIT (Table 2). Hourly IVIT was gradually tapered once blood sugars started to decrease three hours later and IVIT was discontinued six hours after SQ insulin was given, when blood sugar reached 152 mg/dl. Eight hours after receiving SQ insulin, his blood sugar decreased to 90 mg/dl, at which time he was given two ampules of D-50. His blood sugar stabilized, and he continued to do well subsequently and was maintained on a basal-bolus regimen. He was discharged a couple of days later with a basal-bolus insulin regimen.

**Table 2:** Total insulin administered (IVIT and SQ insulin) per day during hospitalization IVIT: intravenous insulin therapy; SQ: subcutaneous

Day since admission	IV insulin (U)	Average glucose (mg/dl)	SQ insulin (U)	Total insulin (U)
1	39	932	20 (Regular Insulin)	59
2	37	467	2 (Insulin lispro)	39
3	90	500	9 (Insulin lispro)	99
4	413	604	32 (Lantus®), 50 (Insulin lispro)	495
5	80	202	20 (Lantus®), 30 (Insulin lispro)	102
6	0	125	20 (Lantus®), 10 (Insulin lispro)	30

## Discussion

It is known that a synergistic relationship exists between COVID-19 infection and hyperglycemia. Patients with diabetes are at an increased risk of severe COVID-19 infection due to their immunocompromised state. COVID-19 can lead to acute hyperglycemic crises in patients with pre-existing diabetes and may sporadically result in new-onset diabetes, occasionally leading to fatal outcomes. 

**Figure 1:**
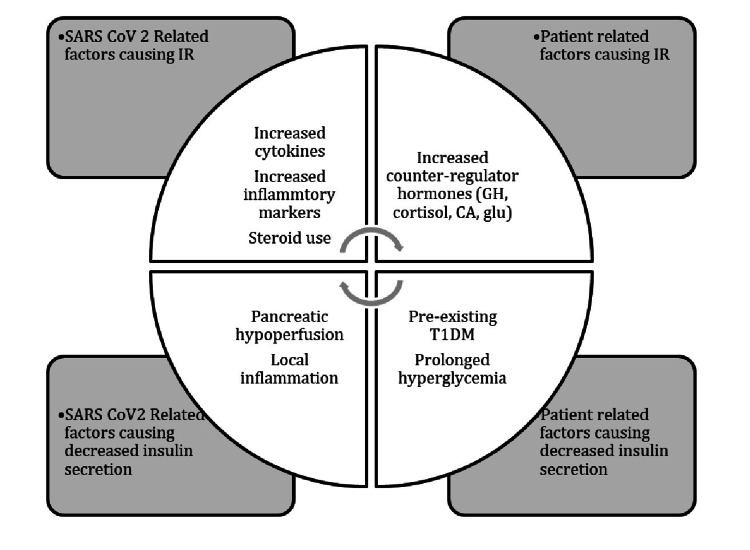
Proposed mechanisms to explain uncontrolled hyperglycemia in patients with COVID-19 infection. COVID-19: coronavirus disease 2019; SARS-CoV-2: severe acute respiratory syndrome coronavirus 2; T1DM: type 1 diabetes mellitus; GH: growth hormone; CA: calcitonin

The pathogenesis of hyperglycemia in the COVID-19 infection is multifactorial. Angiotensin-converting enzyme 2 (ACE 2) is the main entry receptor of SARS-CoV-2 and is expressed in many human cells and tissues, including pancreatic islet cells. SARS-CoV-2 binds to the ACE 2 receptors expressed in the pancreatic islet cells causing transient destruction, and insulin deficiency. COVID-19 infection is accompanied by high levels of inflammatory markers, particularly IL-6 and other acute phase reactants, which could directly lead to the destruction of the pancreatic cells and additionally contribute to insulin resistance while promoting ketogenesis [2]. Some reports also suggest that SARS-CoV-2 can induce adipocyte dysfunction further contributing to insulin resistance and hyperglycemia [3]. Stress hyperglycemia characterized by insulin resistance increased lipolysis and increased circulating free fatty acids appear to play a major role in contributing to hyperglycemia, particularly during the cytokine storm phase. Figure 1 shows a few mechanisms to explain uncontrolled hyperglycemia in patients with COVID-19 infection.

The replication of the virus in monocytes is dependent on denosine triphosphate (ATP) production by glycolysis, explaining the correlation between the degree of hyperglycemia and the severity of COVID-19 infection [4]. Hence, it is unusual to expect severe hyperglycemia with mild COVID-19 infection. Worsening hyperglycemia in the months following COVID-19 infection in patients with pre-existing well-controlled diabetes has also been reported, likely secondary due to a state of insulin resistance and/or insulin deficiency, emphasizing the long-term effects of the virus on glucose metabolism, despite the resolution of the infection [5].

Our patient with pre-existing T1DM was admitted to the hospital with DKA after refusal of insulin and hemodialysis. He was found to be positive for COVID-19 on routine testing, although he was asymptomatic, maintained oxygen saturation, and did not require steroids to treat the infection. Interestingly, he had three admissions within the last six months as well as another admission two weeks after the current presentation with DKA secondary to insulin non-compliance, all of which required IV insulin for <24 hours following which he was transitioned to a basal-bolus insulin regimen with well-controlled glucose levels. The degree of the patient’s hyperglycemia and insulin resistance was out of proportion to his asymptomatic COVID-19 status. He was started on IVIT, despite which he had initial worsening of hyperglycemia and required as high as 500 units of insulin in 24 hours on the fourth day of admission.

Additional workup revealed elevated inflammatory markers, particularly ferritin, fibrinogen, and erythrocyte sedimentation rate (ESR). Counter-regulatory hormones including cortisol and growth hormone were also elevated. This suggests that hyperglycemia could have resulted due to a combination of cytokine-mediated insulin resistance as well an increase in counter-regulatory hormones in the background of complete insulin deficiency as evidenced by undetectable C-peptide levels. Insulin antibodies were also positive, which could have contributed towards hyperglycemia; however, we are unsure if this was a new development or if the patient had pre-existing antibodies as is seen often in patients on long-term insulin. Rapid metabolism of intravenous insulin remains a possible hypothesis as well. 

Hyperglycemia is associated with a poor prognosis in hospitalized patients with COVID-19 infection making it crucial to achieve optimal glycemic control, particularly during the initial stages of the infection to prevent complications including secondary infections, multiorgan failure, ventilator dependence, and mortality [6]. Some patients with severe hyperglycemia in a setting of COVID-19 often require doses as high as 40 units/hour [7,8].

## Conclusions

We presented a rare case of severe insulin resistance in a patient with asymptomatic COVID-19 infection and underlying T1DM, who developed severe insulin resistance with poor response to high doses of IV insulin. He ultimately responded to a combination of high-dose SQ insulin and high doses of IVIT with a resolution of ketoacidosis and hyperglycemia. The proposed mechanisms for hyperglycemia in COVID-19 include stress and steroid-induced hyperglycemia contributing to insulin resistance, as well as the direct effects of SARS-CoV-2 on the pancreatic β cells causing transient destruction and insulin deficiency. The virus is dependent on ATP generated by glycolysis for replication explaining the association between moderate to severe COVID-19 infection and hyperglycemia and, hence, it is unusual to have severe insulin resistance with mild infections. Patients with underlying diabetes with COVID-19 infection require close monitoring since even a mild infection can cause severe hyperglycemia necessitating the need for IVIT.
